# Influence of Metal Artifact Reduction Algorithms on the Detection of Inferior Alveolar Canal Perforations by Zirconia Implants in CBCT Images

**DOI:** 10.1002/cre2.70397

**Published:** 2026-06-30

**Authors:** Parisa Soltani, Mohammad Hossein Manouchehri, Sara Rostamian, Hugh Devlin, Amirhossein Moaddabi, Aida Ekhtiari, Mahsa Aeinehvand, Gianrico Spagnuolo, Reyhaneh Rostamian, Niccolo Giuseppe Armogida, Francesco Riccitiello, Mariangela Cernera

**Affiliations:** ^1^ Department of Oral and Maxillofacial Radiology, Dental Implants Research Center, Dental Research Institute, School of Dentistry Isfahan University of Medical Sciences Isfahan Iran; ^2^ Department of Neurosciences, Reproductive and Odontostomatological Sciences University of Naples ‘Federico II’ Naples Italy; ^3^ Department of Oral and Maxillofacial Surgery, School of Dentistry Isfahan University of Medical Sciences Isfahan Iran; ^4^ Student Research Committee, School of Dentistry Isfahan University of Medical Sciences Isfahan Iran; ^5^ The Dental School The University of Bristol Bristol UK; ^6^ Department of Restorative Dentistry, School of Dentistry Jordan University Amman Jordan; ^7^ Department of Oral and Maxillofacial Surgery, Dental Research Center Mazandaran University of Medical Sciences Sari Iran; ^8^ Department of Oral and Maxillofacial Radiology, Student Research Committee, School of Dentistry Isfahan University of Medical Sciences Isfahan Iran; ^9^ Department of Biomedical Engineering, Science and Research Branch Islamic Azad University Tehran Iran; ^10^ Global Research Cell, D. Y. Patil Dental College & Hospital D. Y. Patil Vidyapeeth, Pimpri Pune India; ^11^ School of Mechanical Engineering, College of Engineering University of Tehran Tehran Iran

**Keywords:** artifact, cone beam computed tomography, dental implant, mandibular canal

## Abstract

**Introduction:**

This study aims to evaluate the efficacy of a metal artifact reduction (MAR) algorithm in detecting contact between zirconia implants and inferior alveolar canal.

**Materials and Methods:**

Twelve zirconia implants were placed in dry human mandibles using surgical guides, in two conditions: 1 mm above and within the canal. cone beam computed tomography (CBCT) was acquired with the MAR algorithm turned off and on. Three observers indicated whether canal damage was present using a 5‐point Likert scale: 1. Definitely no contact, 2. Probably no contact, 3. Uncertain, 4. Probably contact, and 5. Definitely contact. Sensitivity and specificity were calculated and the ROC curve was used to compare the two conditions (*α* = 0.05).

**Results:**

The area under curve (AUC) was higher in MAR On condition for all observers; however, the differences in AUC between the On and Off MAR states were not statistically significant (*p* > 0.05): for observer 1, AUC increased from 0.9916 (Off) to 1.0 (On); for Observer 2, it increased from 0.7666 to 0.8833; and for Observer 3, it increased from 0.95 to 1.0. Observers 1 and 3 displayed 100% sensitivity and specificity regardless of MAR activation, while for Observer 2, diagnostic sensitivity increased from 66.7% to 83.3% with MAR activation, while specificity remained constant at 80% in both conditions.

**Conclusion:**

For the detection of contact of the zirconia implants with the IAC, activating the MAR algorithm slightly increased the diagnostic performance metrics of CBCT images, but the differences were not statistically significant.

**Clinical Relevance:**

Activating the MAR algorithm did not produce a statistically significant improvement in detecting contact between zirconia implants and the inferior alveolar canal.

## 1 Introduction

Titanium implants have garnered more attention in dentistry than other types for treatment of edentulism. With advancements in manufacturing technology and dental materials, zirconia implants have emerged as an alternative due to their tooth‐like color, optimal mechanical properties, favorable biocompatibility, reduced plaque adhesion, and consequently, potentially lower bone resorption (Ji et al. 2024; Shrivastava et al. 2024). Precise placement of dental implants is essential to prevent subsequent complications. During or after implant placement, there is a risk of injuring branches of the trigeminal nerve, such as the inferior alveolar and mental branches (Kämmerer et al. 2024). Inferior alveolar nerve injury is among the most severe complications of dental implant surgery. While some cases are temporary and manageable, others result in permanent damage. According to Libersa et al. (2007), 75% of patients with neurosensory disturbances after implant surgery experienced lasting injuries. While, Renton et al. (2012). reported permanent neuropathy in 27 out of 30 patients, with more than half suffering from ongoing pain and discomfort. Long‐lasting injuries can affect all aspects of life, reduce quality of life, and potentially lead to legal issues for clinicians (Yilmaz et al. 2016). Therefore, the precise diagnosis of injuries to the inferior alveolar nerve resulting from implant placement is of paramount importance.

Radiology is one of the most widely used diagnostic tools in dentistry and plays a crucial role in various stages of implant treatment, including preoperative assessment, treatment planning, implant placement, and follow‐up. Several imaging modalities, such as periapical radiography, panoramic radiography, and cone beam computed tomography (CBCT), are used in different phases of implant‐based treatments (Lam and Mallya 2024). However, two‐dimensional imaging techniques, including periapical and panoramic radiographs, have limitations such as distortion, magnification of structures, anatomical superimposition, and the inability to assess the buccolingual dimensions (Vanderstuyft et al. 2019; Sirin et al. 2020). In contrast, CBCT, as a three‐dimensional modality, provides valuable information throughout implant treatment. Dental implants are composed of high‐density materials, which can lead to artifacts in radiographic images, thereby reducing image quality and potentially compromising the accurate diagnosis of postoperative complications (Soltani et al. 2024a). These artifacts may hinder the precise identification of various complications following implant placement, including damage to vital anatomical structures (Soltani et al. 2024b).

Various methods exist to minimize artifact generation in CBCT images, such as adjusting tube current (mA), increasing voltage (kV), preventing patient movement, and reducing the size and shape of the imaged area. However, artifacts remain a diagnostic challenge, and particularly, reducing or eliminating metal artifacts in CBCT images can prove difficult (Cascante‐Sequeira et al. 2023). A practical approach to eliminating metal artifacts is the use of metal artifact reduction (MAR) algorithms (Mouton et al. 2013). Zirconia implants generate more artifacts than titanium implants due to their higher atomic number, making it more challenging to accurately visualize adjacent structures (Soltani et al. 2024a). Given the additional costs associated with MAR algorithms and their variable efficacy, sometimes even negatively impacting diagnostic accuracy, their effectiveness must be evaluated for specific clinical applications.

In 2023, de Freitas et al. (2023) evaluated the influence of MAR in the diagnosis of dental implant contact with the IAC in CBCT images in an ex vivo study on 10 dry human mandibles. They concluded that, due to the limited efficacy of MAR, it should not be used when conducting CBCT scans for the evaluation of contact between the implant and the mandibular canal. In 2024, Soltani et al. (2024b) conducted a study to evaluate the effectiveness of the MAR algorithm in diagnosing injuries caused by titanium implants in the inferior alveolar canal (IAC). The findings indicated that while the MAR algorithm had some positive effects, its application did not significantly improve the diagnostic accuracy of CBCT images. In another study in 2024, Capel et al (2024) conducted an experimental study to assess the qualitative impact of the MAR algorithm on the diagnosis of titanium implant‐induced damage to the IAC. The findings indicated that employing the MAR algorithm at its high intensity led to a decrease in diagnostic accuracy compared to its use at moderate intensity or when turned off.

Although several studies have evaluated the diagnostic impact of MAR algorithms for titanium implants, the overall evidence consistently shows limited or inconsistent benefit, and in some cases even reduced diagnostic accuracy. These findings raise important questions about whether MAR should be routinely applied for implant‐related assessments. However, zirconia implants differ fundamentally from titanium in their physical properties and produce substantially more severe artifacts, suggesting that conclusions drawn from titanium‐based studies cannot be directly extrapolated. Therefore, it remains unclear whether MAR algorithms might perform differently when applied to zirconia‐induced artifacts. Addressing this gap is essential for establishing evidence‐based imaging protocols for zirconia implant cases. A review of the existing literature reveals no studies assessing the performance of MAR algorithms in detecting contact between zirconia implants and the IAC. Therefore, this study aimed to evaluate the effectiveness of MAR algorithms in detecting zirconia implant contact with the mandibular canal in CBCT images.

## 2 Materials and Methods

Three unidentified dry human mandibles from the archive of the Department of Anatomy, Isfahan University of Medical Sciences, were used in this study. This study was ethically approved by the institutional Research Ethics Committee (number: IR.MUI.REC.1404.004; approval date: 30/4/2025).

### 2.1 Samples Size Calculation

To compare the mean diagnostic accuracy of CBCT images with and without the activation of the MAR algorithm, the required sample size was calculated using GPower software. Considering a Type I error of 5%, a power of 90%, and an effect size of 1.155 extracted from a similar study by (de Freitas et al 2023) based on Table 1 (comparison of accuracy between 4 mA MAR OFF and 4 mA MAR On), the required sample size was determined to be 10 implants. Therefore, accounting for a 20% dropout (or failure) probability, the final sample size was set at 12 implants.

**Table 1:** Diagnostic values of CBCT images with/without MAR activation for each observer.

Observers	MAR condition	AUC (95% CI)	Sensitivity	Specificity	*p*‐value	AUC difference
1	On	1 (0.78, 1.00)	1	1	0.95	0.0083
	Off	0.99 (0.77, 1.00)	1	1		
2	On	0.88 (0.60, 1.00)	0.83	0.8	0.94	0.1166
	Off	0.77 (0.47, 1.00)	0.67	0.8		
3	On	1 (0.78, 1.00)	1	1	0.70	0.05
	Off	0.95 (0.72, 1.00)	1	1		

Abbreviations: AUC, area under curve; CI, confidence interval; MAR, metal artifact reduction

### 2.2 Fabrication of Surgical Guides

CBCT images were acquired from the mandibles using a Papaya 3D scanner (Genoray, Seongnam‐Si, Korea) with the following exposure parameters: 85 kVp, 8 mA, a voxel size of 140 µm, a field of view of 11 cm × 11 cm, and a scan time of 14.5 s. The DICOM files of these images were exported and forwarded for surgical guide design.

**Figure 1:**
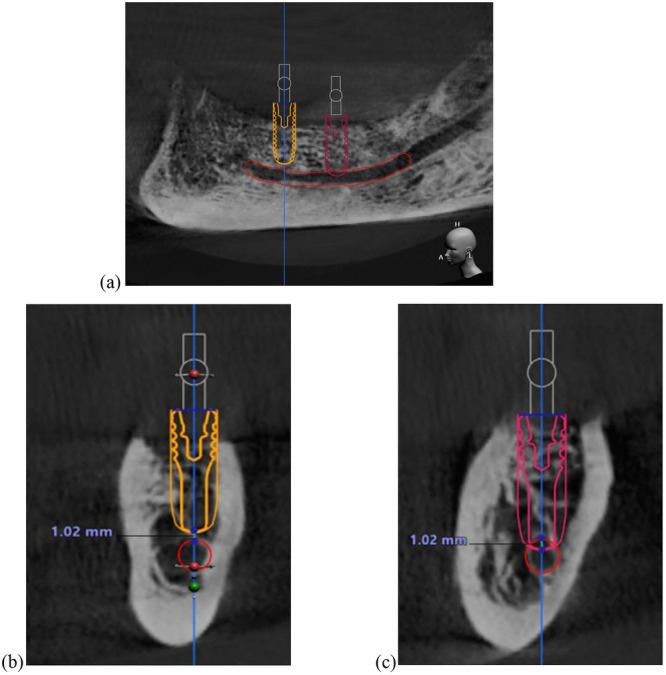
Virtual placement of implants prior to surgical guide fabrication (a) in tangential and (b) and (c) cross‐sectional views.

For the fabrication of the surgical guide, a laboratory technician segmented the DICOM file using Materialise Mimics software (Materialise NV, Leuven, Belgium) to convert the images into the STL (Standard Triangle Language) format. Subsequently, using 3Shape software (Copenhagen, Denmark), 12 White Sky zirconia implants (Bredent, Senden, Germany) with dimensions of 12 × 4 mm were virtually positioned at the sites corresponding to the first and second molars of each hemi‐mandible in two configurations: one with the implant placed 1 mm above the IAC and the other with the implant positioned 1 mm within the IAC, in such a way that the canal's cortex was perforated (Figure 1). Therefore, 4 implants (2 implants per side) were placed in each of the 3 mandibles. Finally, the surgical guide was produced via 3D printing (Asiga, Sydney, Australia).

### 2.3 Placement of Dental Implants

**Figure 2:**
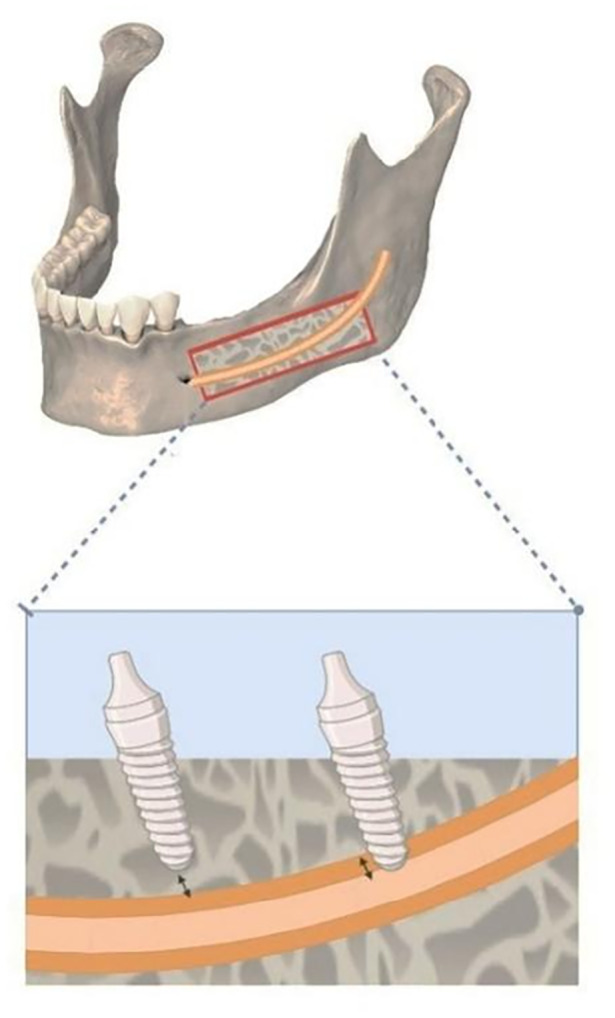
Schematic demonstration of the position of implants in relation to the inferior alveolar canal (Created in https://BioRender.com).

After the fabrication of the surgical guide, 12 zirconia implants (White Sky, Bredent, Senden, Germany; 4 × 12 mm) were placed in the indicated region using the corresponding implant‐guided surgery system according to the manufacturer's instructions (Figure 2). After completing the imaging procedures, the spatial relationship between each implant and the IAC was confirmed by surgically opening a buccal window, allowing direct visualization of the canal and the implant tip.

Following the surgical procedure, the mandibles were subsequently covered with a 0.5‐cm‐thick layer of acrylic resin to simulate soft tissue (Soltani et al. 2024c).

### 2.4 CBCT Image Acquisition

CBCT scanning was performed using a Papaya 3D scanner (Genoray, Seongnam‐Si, Korea) with the parameters of 85 kVp, 8 mA, a voxel size of 140 µm, a field of view of 11 cm × 11 cm, and a scan time of 14.5 s. For initial image preparation and review, the native Theia software (Genoray, Seongnam‐Si, Korea) was used. In this software, after the scan, the images were saved in two modes: one without activating the artifact reduction algorithm (referred to as SMARF in the software) and the other with the algorithm activated.

### 2.5 CBCT Image Evaluation

After a calibration session on images of two zirconia fixtures from a pilot study, all images were evaluated by three observers: observer 1: oral and maxillofacial radiologist (P.S. with 9 years of experience); observer 2: oral and maxillofacial surgeon (M.H.M. with 9 years of experience); observer 3: senior oral and maxillofacial radiology resident (A.E. with 3 years of experience). The evaluations were conducted in a dimly lit, quiet room on an identical monitor. The observers were blinded to both the presence or absence of damage in the images and the status of the MAR algorithm (activated or deactivated), and the images were presented in a random order. The observers had the freedom to choose their preferred view and could utilize all available software options (zoom, contrast adjustment, brightness adjustment, etc.) without any time limitations for each individual analysis. For each implant, the observers assigned a score to the relationship of the implant tip and the IAC based on a 5‐point Likert scale as follows: 1. Definitely no contact, 2. Probably no contact, 3. Uncertain, 4. Probably contact, and 5. Definitely contact. For each observer, only one viewing session, lasting up to 2 h, was scheduled per day. To calculate intraobserver agreement, 20% of images were analyzed after 1 month by each observer.

### 2.6 Statistical Analysis

Scores 1 and 2 were classified as indicating “no contact” between the dental implant and the MC, while scores 4 and 5 indicated “contact.” Cases with a score of 3 (“uncertain”) were excluded from the data analysis. In this study, no observer used score 3. To describe the variables in the present study, the percentage index was used. Sensitivity and specificity were calculated for each observer. Diagnostic accuracy for different conditions was determined using the area under the receiver operating characteristic (ROC) curve. To compare the area under the curve between the two conditions, MAR On and MAR Off, for each observer, a paired *t*‐test was employed. Interobserver and intraobserver agreements were assessed using the kappa coefficient. Statistical analyses were performed using SPSS software (IBM Statistics, Armonk, NY, United States) with a significance level set at 0.05.

Reporting of the present manuscript complies with Strengthening the Reporting of Observational Studies in Epidemiology (STROBE) guideline.

## 3 Results

**Figure 3:**
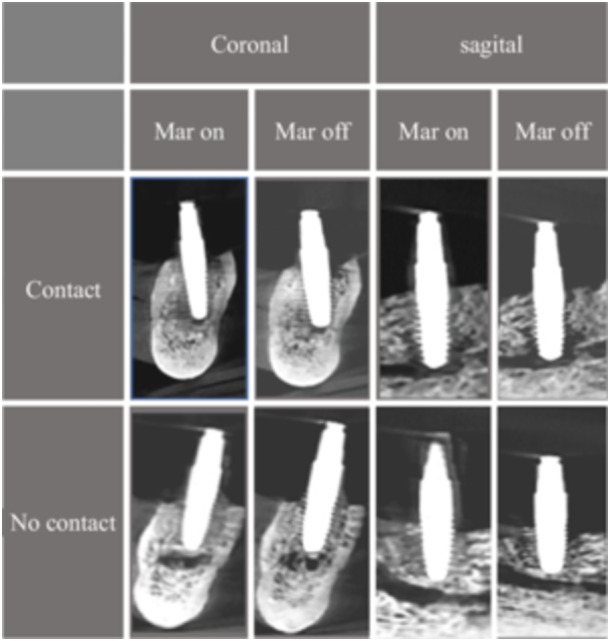
Tangential and cross‐sectional CBCT views of a zirconia implant with and without contact with the IAC in MAR Off and On conditions.

Figure 3 shows resultant CBCT images of zirconia implants with and without contact to the IAC.

Data analysis showed that the intraobserver agreement for each observer was at an excellent level. The kappa coefficient for observers 1 and 2 was 0.981 and for observer 3, it was 1. This indicates the accuracy and consistency of their individual assessments. In addition, the results showed that the degree of interobserver agreement was almost excellent (0.821), indicating a significant alignment in their opinions and assessments.

**Figure 4:**
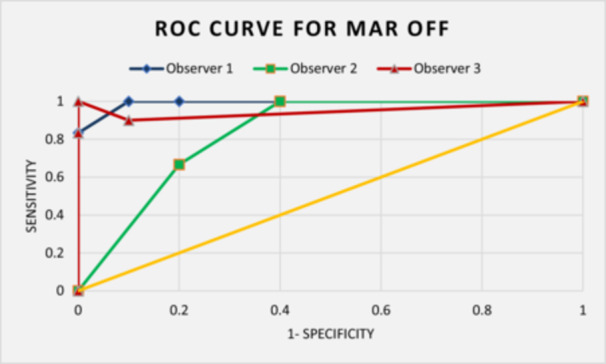
Comparison of the area under the curve in the On and Off mode of the MAR algorithm.

**Figure 5:**
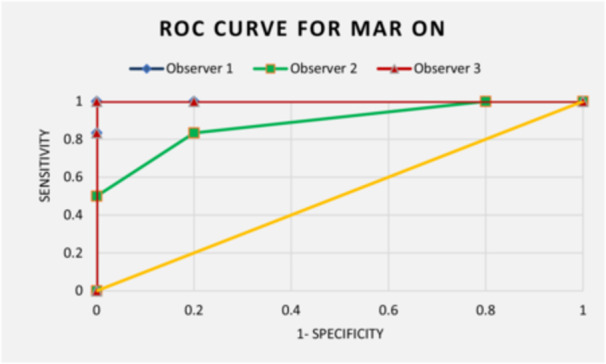
Comparison of the area under the curve in the active mode of the MAR algorithm.

Table 1 and Figures 4 and 5 provide the diagnostic performance values for each observer. None of the observers assigned a score of 3 (uncertain) to any case. Based on the performed analyses, for observer 1 both the sensitivity and specificity were equal to 1 in MAR On and Off conditions. The area under the ROC curve (AUC) in MAR Off condition was 0.99, which is slightly lower than that in the MAR On state (AUC = 1). No statistically significant difference was found between the AUCs in the active and inactive states of the MAR (*p* = 0.953).

For observer 2, the diagnostic sensitivity was 0.83 in the active state and 0.67 in the inactive state. The diagnostic specificity of the MAR algorithm was 0.8 in both MAR Off and On conditions. Moreover, the AUC in the MAR inactive state was 0.77, which is lower than that in the active state (AUC = 0.88). No statistically significant difference was observed between the AUCs of the active and inactive states of the MAR algorithm (*p* = 0.944).

For observer 3, both sensitivity and specificity were equal to 1 regardless of whether the MAR algorithm was On or Off. In addition, the AUC in the MAR Off condition was 0.95, which is lower than the value of 1 observed in the MAR On state. No statistically significant difference was detected between the AUCs in the MAR On and Off conditions (*p* = 0.702).

## 4 Discussion

Based on the results of the present study, although the area under the ROC curve was higher for all three observers when the MAR algorithm was active compared to when it was inactive, this difference was not statistically significant. Additionally, activating the MAR algorithm led to an increase in sensitivity and specificity for only one observer.

Renton et al. (2011), in 2011, reported 183 iatrogenic injuries to the branches of the trigeminal nerve, of which 90 were related to inferior alveolar nerve injuries. They also found that over 70% of patients experienced a combination of neuropathic pain, numbness, altered sensation, and paresthesia of the chin and lip, symptoms that negatively affect various functions such as facial expression, eating, and drinking. The treatment of these issues is time‐consuming and challenging, and it has been reported that more than 60% of patients with inferior alveolar nerve injuries do not achieve complete recovery (Renton and Yilmaz 2012). Therefore, an accurate diagnosis of the cause and nature of the injury is of crucial. Although CBCT, due to its three‐dimensional image reconstruction capability, is considered superior to panoramic radiography in implant treatment planning, its susceptibility to metal artifacts might limit its diagnostic potential in cases where an object of high atomic number, e.g. a dental implant, is located in the imaging field (Lam and Mallya 2024; Soltani et al. 2024a). Therefore, the attempt to reduce or eliminate these artifacts is an ever‐growing field of research in radiology (Selles et al. 2023). In addition, the effectiveness of MAR algorithms in enhancing diagnostic image quality remains under debate.

To the best of the authors' knowledge, no previous study has been performed to evaluate the impact of the MAR algorithm on the interpretation of CBCT images for zirconia implant injuries to the IAC; only three studies have assessed the effect of MAR on CBCT image interpretation in cases of titanium implant injuries to the IAC. Overall, the results of these three studies indicate that the use of the MAR algorithm does not lead to a significant improvement in the diagnostic accuracy for titanium implants. Despite differences in implant materials, these findings are generally consistent with the results of the present study.

In the study by de Freitas et al. (2023) in 2023, 18 titanium implants were positioned using surgical guides in dry human mandibles in two positions of with and without contact with the IAC. The mandibles were imaged with two CBCT scanners, Eagle 3D and OP 300, with and without activation of the MAR algorithm. Their findings indicated a considerable negative effect of MAR activation on the sensitivity for dento‐maxillofacial radiologists. In the present study, the use of MAR led to an increase in some of the diagnostic performance metrics of CBCT images but this improvement was not significant. The CBCT scanners and MAR algorithms used in the two studies were not similar. Moreover, in the study of de Freitas et al. the time of activation of MAR is not mentioned. In the present study, the CBCT scanner that was used only allows MAR activation after image acquisition. In addition, in the present study, zirconia implants were used, while de Freitas et al. placed titanium implants.

In the study by Soltani et al. (2024b) in 2024, with the aim of evaluating the diagnostic performance of the MAR algorithm for titanium implant‐induced injuries to the IAC, 36 titanium implants were placed in sheep hemi‐mandibles in three positions: pilot drill injury to IAC, penetration injury to IAC, and control. Implants were imaged by the Galileos CBCT scanner with and without activation of MAR after exposure. Similar to the findings of the present study, the use of MAR did not lead to a significant improvement in the diagnostic accuracy of CBCT images.

In 2024, Capel et al. (2024) inserted 18 titanium implants in dried mandibles at 0.5 mm superior to the IAC and 0.5 mm inside the IAC. CBCT scans were taken by the Eagle 3D scanner with different levels of MAR (off, medium, and high). Their results showed that specificity and area under ROC curve decreased significantly when the MAR level was high compared with MAR‐medium and MAR‐off. This finding can be attributed to different CBCT scanners and MAR algorithms, as well as different implant materials (zirconia in the present study, titanium in the study of Capel et al.). Additionally, the CBCT scanner used in the present study did not have the option for adjusting the MAR level. Furthermore, while the study of Capel et al. does not specify when the MAR algorithm was activated, the CBCT scanner used in the present study permits MAR to be activated only after image acquisition.

Many studies have investigated the optimal distance between an implant and the canal. Başa et al. examined the thickness of the superior wall of the inferior alveolar canal on CBCT images and concluded that the canal's thickness should be assessed before implant placement (Başa and Dilek 2011). In 2021, Froum et al. (2021)., after evaluating 101 implants with less than 2 mm between the implant tip and IAC, found that a distance of 2 mm from the canal is an overestimation, and an average distance of 0.75 mm from the canal border to the implant has been suggested. However, considering the differences in position, angle, and surgical conditions, it is prudent to maintain a distance of 2 mm from the canal during implant placement.

In interpreting the observer‐specific differences, particularly the greater improvement observed for Observer 2, the potential influence of professional background and experience warrants consideration. Despite their heterogeneous clinical profiles, all observers were experienced and formally trained in interpreting CBCT images for implant dentistry, and they underwent a structured calibration process before image evaluation and were blinded to the experimental conditions. The very high intraobserver agreement (*κ* = 0.981 for Observers 1 and 2; *κ* = 1.0 for Observer 3) and the excellent interobserver agreement (*κ* = 0.821) indicate that their assessments were highly consistent despite differences in specialty. Nevertheless, the modest improvement seen only in Observer 2 with MAR activation may reflect subtle variations in diagnostic experience or familiarity with CBCT artifact patterns. Because the study was not powered to formally test observer background as an interaction factor, this hypothesis cannot be statistically confirmed. However, it highlights an important avenue for future research: larger studies incorporating observer expertise as an analytical variable may clarify whether MAR algorithms preferentially benefit clinicians with intermediate experience levels, while experts may already perform near ceiling regardless of MAR activation. In addition, two observers achieved perfect diagnostic performance, which may reflect a ceiling effect partly influenced by the limited number of samples, despite the use of an a priori sample size calculation. While this does not compromise the validity of the findings, it may reduce sensitivity to detect subtle differences between MAR and non‑MAR conditions and limit generalizability to clinicians with varying levels of experience.

In the present study, the diagnostic efficacy of activating the MAR algorithm was evaluated for detecting two types of simulated injuries to the inferior alveolar canal. These defects were created at approximately the 1 mm limit of the canal, reflecting clinical scenarios in which minor miscalculations of the available bone height during preoperative radiographic assessments often lead to such injuries. One limitation of this study is its ex vivo design; however, using dry human mandibles with a digital surgical guide design for precise implant insertion provides a valuable simulation of clinical conditions. Additionally, only one CBCT scanner which permits MAR to be activated only after image acquisition was used. Because the findings are based on a single CBCT device with a specific MAR implementation, the generalizability of the results to other scanners or MAR algorithms is inherently limited. Investigating the effects of other MAR algorithms, particularly artificial intelligence‐driven methods, offers an interesting avenue for future research.

## 5 Conclusion

For the detection of contact of the zirconia implants with IAC, activating the MAR algorithm slightly enhanced the diagnostic potential of CBCT images; however, MAR activation did not significantly improve diagnostic accuracy under the tested conditions.

## Author Contributions

Conceptualization: Parisa Soltani, Hugh Devlin, Sara Rostamian. Methodology: Parisa Soltani, Mohammad Hossein Manouchehri, Sara Rostamian, Aida Ekhtiari, Mahsa Aeinehvand. Software. Data curation: Parisa Soltani, Amirhossein Moaddabi. Investigation: Sara Rostamian, Aida Ekhtiari. Validation: Mohammad Hossein Manouchehri. Formal analysis: Amirhossein Moaddabi, Hugh Devlin, Gianrico Spagnuolo, Niccolo Giuseppe Armogida, Reyhaneh Rostamian. Supervision: Parisa Soltani. Funding acquisition: Mohammad Hossein Manouchehri. Visualization. Project administration: Gianrico Spagnuolo, Niccolo Giuseppe Armogida. Resources: Sara Rostamian. Writing – original draft: Parisa Soltani, Mohammad Hossein Manouchehri, Sara Rostamian. Writing – review and editing: Amirhossein Moaddabi, Hugh Devlin, Aida Ekhtiari, Mahsa Aeinehvand, Gianrico Spagnuolo, Niccolo Giuseppe Armogida.

## Ethics Statement

This study was approved by the Research Ethics Committee Isfahan University of Medical Sciences, under registration number: IR.MUI.REC.1404.004.

## Conflicts of Interest

The authors declare no conflicts of interest.

## Data Availability

The data that support the findings of this study are available from the corresponding author upon reasonable request.
